# Adherence to antiretroviral therapy among HIV infected pregnant women in public health sectors: a pilot of Chilenje level one Hospital Lusaka, Zambia

**DOI:** 10.11604/pamj.2020.35.49.20078

**Published:** 2020-02-19

**Authors:** Moses Mukosha, Grace Chiyesu, Bellington Vwalika

**Affiliations:** 1 Department of Pharmacy, University of Zambia, Lusaka, Zambia; 2 Mosi-o-Tunya University of Science and Technology, Lusaka, Zambia; 3 Faculty of Pharmacy Nutrition and Dietetics, Apex Medical University, Lusaka, Zambia; 4 Department of Obstetrics and Gynecology, University of Zambia, Lusaka, Zambia

**Keywords:** HIV, adherence, Zambia, antiretroviral therapy (Option B+)

## Abstract

**Introduction:**

regular use of Antiretroviral Therapy (ART) in pregnancy and breastfeeding reduces the odds of Mother-to-Child HIV Transmission (MTCT). However, adherence to ART is critical for MTCT to be successful. The present study investigated factors that influence adherence to ART among HIV infected pregnant women in Zambia.

**Methods:**

a cross-sectional study design was conducted involving 71 HIV infected pregnant women who were advised to join the Prevention of Mother-to-Child HIV Transmission (PMTCT) program during their routine Antenatal clinic (ANC) visit and were on ART for more than six months. We used the Medication Possession Ratio (MPR) to quantify adherence levels. We used logistic regression to establish factors that influence adherence to ART.

**Results:**

a total of 71 HIV infected pregnant women with a median age of 27years (IQR, 25-30) were enrolled in the study. There was evidence of a difference in adherence levels between pregnant women above 30 years and ones between 15 years and 30 years (P<0.001). Median adherence levels in this group were found to be at 96%(IQR 89-97). The main predictor of adherence in this population was marital status (being on separation) and age. The women who were on separation were 0.14 times less likely to adhere to option B+ compared to married women.

**Conclusion:**

adherence to option B+ among pregnant women is low. Adherence was significantly influenced by marital status (being on separation) and age. Efforts to improve adherence should be directed towards women on separation and young adults (< 30 years of age).

## Introduction

According to the World Health Organisation (WHO) by the end of 2016, about 19.5 million people were receiving ART worldwide [1]. ART program in Zambia has expanded rapidly with over 120,000 people receiving ART at over 160 sites throughout the country by 2017. Zambia has scaled up the Option B+ program countrywide with the focus on adherence and universal access to treatment [2]. Under Option B+, HIV is detected in women during routine testing in antenatal care, when they are asymptomatic, and these women start medication shortly after that [3, 4]. The women under Option B+ in Zambia receive a daily fixed-dose combination of efavirenz, tenofovir, and lamivudine [5]. They collect new medication at each monthly visit for the first six months of treatment, and every 2-3 months after that [5, 6]. The ministry of health (MoH) adopted 2014, Joint United Nations Programme on HIV/ AIDS (UNAIDS) ambitious global targets, including “90-90-90” by the year 2020 which means that 90% of all people with diagnosed HIV infection will receive sustained ART [7]. This approach will no doubt bring about a reduction in new infections as most patients will have a suppressed viral load. Despite the tremendous successes this approach promises, it is likely that as the number of people on treatment and average duration of therapy increases, there will be an increase in levels of HIV drug resistance (HIVDR) as a consequence of poor adherence [8–11]. Therefore, improving adherence is critical to maintaining improved patient outcomes, protecting investments, and guaranteeing the long term sustainability of care and treatment programmes in Zambia and other Low Middle-income Countries (LMIC) [5,12,13]. The cost of poor adherence has far-reaching consequences which cannot be underestimated. Poor adherence is associated with poor virological outcomes and increased oxidative stress which can result in preeclampsia [14–19], increased mortality [20–22], reduced durability and effectiveness of regimens [23,24] developing drug resistance [25–27]. Drug resistance is more complicated to deal with, and costlier in that alternative drugs are more expensive, have more side effects, and are not as widely available as 1st line drugs [28–30]. It is not clear in current medical literature the adherence levels to option B+ and associated factors related to pregnant women attending ANC at 1^st^ level Chilenje Hospital in Lusaka, Zambia. Therefore, this study was set out to establish adherence levels and associated factors to better inform policy guidelines on simple, cost-effective interventions that would improve ART and maternal outcomes at 1^st^ level Chilenje Hospital and other hospitals in Zambia.

## Methods

The pregnant and breastfeeding women with HIV infection in Zambia are offered ART with same-day initiation regardless of CD4 count (Option B+) [12]. Because we did not have access to viral load information to validate the adherence threshold, we used a more stringent measure of adherence like one used in Zimbabwe [31] and Ethiopia [32] but different from a study done in Malawi [33]. For this study, patients with Medication Possession Ratio (MPR) >95% were considered “optimally adherent”; those with MPR of <95% “poorly adherent.” We calculated MPR by dividing the number of days late for pharmacy refills by total days on therapy and then subtracted that percentage from 100%. The figure that we get represents the percentage of days that a patient is known to have medication on hand [23]. Whereas it is possible that some women skipped days and then resumed pill taking, for simplicity we presumed that women took medication on successive days and then stopped similar to a study done in Ethiopia [32]. The recommended first-line ART regimen in Zambia is the three drug combination of Tenofovir, Lamivudine and Efavirenz. In Zambia, Option B+ protocol specifies ART medication pick-up visits 2 weeks post initiation, and afterwards monthly during the ANC and postnatal periods. We measured stigma using a six-item Internalized AIDS-Related Stigma Scale which previously showed high levels of reliability and validity in an African setting (Kalichman *et al*. 2005, 2009; Tsai *et al*. 2013). A binary response scale was used, that is, “agree” versus “disagree”. We summed the scale scores to obtain a composite stigma score ranging from 0 to 6, with higher scores indicating more internalized stigma.

**Study setting:** the study was conducted at Chilenje First Level Hospital in Lusaka, Zambia. This facility has one of the largest numbers of people living with HIV, the majority of whom are women enrolled in the ART clinic. The hospital is an entry point of care and is located in a densely populated area with both low and middle-income class. This study included pregnant women on ART for more than 6 months and was recruited in a simplified test and treat policy (Option B+) Program. Option B+ was adopted by Zambia and is now recommended by the World Health Organization (WHO) [6]. Excluded were women who did not give fully informed consent.

**Statistical methods:** for descriptive statistics, the median (interquartile range (IQR<)) for continuous values (i.e. age (years)), was calculated after testing for the assumption of normality using Shapiro-Wilk W-test and graphically qq-plots. Categorical variables were summarised using frequencies and their respective percentages. For the comparison of baseline characteristics, we used Fisher’s exact test after testing for the assumptions of a chi2 test (all of the frequencies in cell should be > 5). We used the investigator-led best model selection to arrive at best predictors of adherence in a multiple logistic regression analysis. In this multivariable regression analysis, we included all covariates reported to be associated with adherence [34–37]. The significance level in this study was set at alpha 0.05 and 95% confidence intervals. Data were analysed using Stata/FC version 15 (stata Corporation, Texas, TX, USA).

**Ethical consideration:** ethical approval was obtained from ERES CONVERGE IRB (approval number 201708026) and written permission to conduct this study from Chilenje level one Hospital was sought from the Lusaka District Health Office Director, Zambia. Before the collection of information, informed consent was sort from the pregnant women who were willing to participate in the study. All details of the research, such as the importance the research has to the health of both the mother and the unborn child was explained to participants. The confidentiality of all records was safeguarded to the extent legally possible, study data, and questionnaire forms are coded by numbers only. Databases are password protected.

## Results

**Figure 1:**
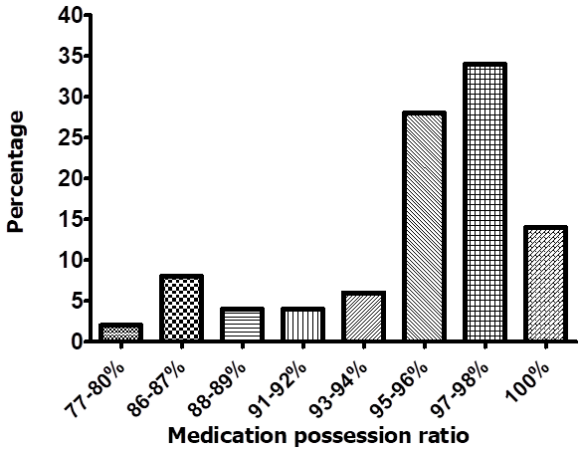
Distribution of adherence to ART over the period of option B plus during pregnancy for the pregnant women retained in care for PMTCT

A total of 71 HIV infected pregnant women with a median age of 27years (IQR, 25-30) were enrolled in the study. There was evidence of a difference in adherence levels between pregnant women above 30 years and ones between 15 years and 30 years (P<0.001). The majority of the participants had attained secondary level education 37/71 (52.1%) and at least 15/71 (21.1%), 19/71 (26.8%) attained primary and tertiary level education, respectively. When stratified according to occupation 12/71 (16.9%) of the participants were in formal employment while 45/71 (63.4%) were in informal sector and 15/71 (21.1%) were unemployed. Furthermore, the majority of the participants were married 41/71 (57.7%), while the rest were either single 11/71 (15.5%), separated 12/71 (16.9%) or widowed 6/71 (8.5%). There was no evidence of a difference in adherence when compared by the level of education (P=0.659), occupation (P=0.327) and marital status (P=0.353) though we could not rule out chance finding. The comparison of adherence with baseline characteristics is shown in Table 1. Figure 1 shows the distribution of adherence to ART over the period of option B+ during pregnancy for the women retained in care for PMTCT at this hospital. The majority of the women showed optimal adherence compared to those who did not. From a total of 71 pregnant women 58/71 (81.7%) demonstrated optimal adherence while 13/71 (18.3%) show poor adherence to ART.

**Table 1:** Baseline characteristics and adherence among participants

Characteristic	Adherent MPR >95%, n=58	Non-adherent MPR <95%, n=13	*P*-value
**Age (years)**			
15-30	47(82.5)	10(17.5)	<0.001
>30	11(78.6)	3(21.1)	
**Education level**			
Primary	12(80)	3(20)	
Secondary	29(78.3)	8(21.6)	0.659
Tertiary	17(89.5)	2(10.5)	
**Occupation**			
Formal	9(75)	3(25)	
Informal	39(86.7)	6(13.3)	0.327
Unemployed	10(71.4)	4(28.6)	
**Marital status**			
Married	33(78.6)	9(21.4)	
Single	11(100)	0	
Separated	9(75)	3(25)	0.353
Widowed	5(83.3)	1(16.7)	

Values are percentages (frequencies), P-values from fisher’s exact test, MPR-Medication possession Ratio

**Adherence to option B plus among pregnant women at Chilenje 1^st^ level hospital:** the crude and adjusted logistic regression model showed participants older than 30 years were 1.1 times more likely to adhere to ART (AOR: 1.10, 95% confidence interval (95% CL): 0.92, 1.30) compared to the ones below this age accounting for other covariates though this was not significant. The pregnant women who were on separation (AOR: 0.14, 95% CL, 0.02, 0.75), single (AOR: 0.23, 95% CL, 0.03, 1.56) and widowed (AOR: 0.18, 95% CL, 0.02, 1.67) were less likely to adhere compared to the married participants though we could not rule out chance finding for the single and the widowed participants. Levels of education showed those who have been to secondary (AOR: 0.89, 95% CL, 0.10, 8.01) and tertiary (AOR: 0.28, 95% CL, 0.04, 2.25) had a reduced odds of adherence compared to the ones who have attended primary school level. No evidence of a difference was observed between the categories of occupation though the participants in informal sector were 1.08 times more likely to adhere compared to the ones in formal sector. Being unemployed was associated with reduced odds (AOR: 0.41, 95% CL, 0.07, 2.20) of adherence compared to being employed in a formal sector. Results of the logistic regression model are shown in Table 2.

**Table 2:** Factors associated with adherence among HIV+ pregnant women at Chilenje Hospital

Characteristics	Crude OR (95% CL)	Adjusted OR (95% CL)
**Age**		
15-30	1	1
>30	1.10(0.95, 1.25)	1.10(0.92, 1.30)
**Marital status**		
Married	1	1
Single	0.36(0.07, 1.83)	0.23(0.03, 1.56)
Separated	0.14(0.03, 0.59)	0.14(0.02, 0.75)
Widowed	0.27(0.04, 1.88)	0.18(0.02, 1.67)
**Stigma**		
Yes	1	1
No	1.11(0.35, 3.50)	1.41(0.33, 6.04)
**Education level**		
Primary	1	1
Secondary	0.56(0.10, 2.99)	0.89(0.10, 8.01)
Tertiary	0.33(0.06, 1.97)	0.28(0.04, 2.25)
**Occupation**		
Formal	1	1
informal	1.08(0.20, 5.92)	0.77(0.11, 5.51)
Unemployed	0.29(0.08, 1.06)	0.41(0.07, 2.20)

OR-Odds Ratio, 95% CL- Confidence interval

## Discussion

The aim of this study was to assess the levels of adherence to option B+ among pregnant women attending antenatal clinic at a public health sector hospital setting. Adherence levels at Chilenje level 1 hospital was found to be at 81.7%. This is a suboptimal adherence based on the Medication possession ratio (classification of adherence levels) that this study adopted. In Malawi with a setting similar to Zambia, they reported that about 70% of the Option B+ patients retained after 2 years adhered adequately (≥90%), but only about a third of women maintained adequate adherence at every visit during the first 2 years of ART [33]. On the other hand in Zimbabwe a study on patterns of HIV Care Attendance and Adherence to ART Among Pregnant and Breastfeeding Women reported that one year after initiation; less than half of the participants (39.1%) were adherent to option B+ [33]. This was similar to a recent study in neighbouring Tanzania, where they found low adherence to option B+ of 26.3% and 61.1% among respondents residing in urban and rural areas, respectively [34]. In South Africa, on the other hand, in a study done by Adeniyi *et al*.[37] reported a high proportion (69.0%) of women with perfect adherence. The observed differences in adherence reported in the region could be attributed to differences in health care services, HIV prevalence and also country set up (rural vs. urban population). The current study is a pilot and mainly hypothesis generation and may not conclusively compare with similar studies that used larger samples. However it gives an insight in the Zambian set up and also what can possibly be expected should we carry a similar study on a larger scale in this setting. Furthermore, the results showed evidence of a difference in adherence levels between pregnant women above 30 years when compared to the ones between 15 years and 30 years. Results of the multivariable logistic regression showed that participants older than 30 years had a 10% increased chance of adhering to option B+ compared to the ones below this age accounting for marital status, occupation, stigma, and level of education though we could not rule out random chance finding.

The effect size may not have been detected, possibly due to the small sample (type II error). Although the sample size was small to lead to firm conclusions, the results are hypothesis-generating and support other similar studies. Haas *et al*.[33] found that being young was one of the risk factors of inadequate adherence in addition to receiving care at district hospitals or health centers. Additionally, in Malawi Erlwanger *et al*.[31] found that compared with adherent patients, patients who were non-adherent or dropped by day 360 were younger, more likely to be in their first pregnancy, and single, characteristics which were strongly associated with participants age. We further noticed that occupation, level of education, and marital status was not significantly different among pregnant women who were adherent compared to those who were not. However, being on separation reduced the odds of adherence to option B+ by 0.14 times compared to married women. Similarly, a study by Zacharius *et al*.[34] found that women with male partners’ support in option B+ PMTCT were 3.51 times more likely to have good adherence than those without. On the other hand, those who attended secondary and tertiary schools were less likely to adhere to option B+ compared to the ones who attended primary school level. On the contrary, no evidence of a difference was observed between the categories of occupation though the participants in informal sector had an 8% chance of adhering when compared to the ones in formal sector. The participants who were unemployed were less likely to adhere to treatment when compared to those who were employed in a formal sector. With the increase in the number of pregnant women who are put on ART there is likely going to be an increase in incidences of drug resistance as a consequence of poor adherence. It is therefore important that efforts are targeted to address the challenges of non-adherence so that optimal drug therapy is sustained in this setting.

## Conclusion

Adherence to option B+ among pregnant women at Chilenje level one hospital is generally suboptimal. Adherence was significantly predicted by marital status (being on separation) and influenced by age. Efforts to improve adherence should be directed towards women on separation and young adults (< 30 years of age). However more information in bigger studies is needed to confirm these findings.

### What is known about this topic

- Adherence is key to optimal drug therapy and favourable therapeutic outcomes;
- Adherence is a challenge in resource limited setting;
- Option B+ has been shown to reduce HIV transmission of Mother to Child in other settings.

### What this study adds

- Option B+ is new in the Zambian settings and adherence levels are still not well understood, the results are hypothesis-generating and can be used to plan for bigger studies;
- Age is a predictor of adherence in this setting;
- It also provides new insights in methodological challenges with regards studies in pregnant women HIV medication adherence.

## Competing interests

The author declares no competing interests.
